# Supplementary material for: The interactome of intact mitochondria by cross-linking mass spectrometry provides evidence for coexisting respiratory supercomplexes
Source: Mol Cell Proteomics. 2017 Dec 8;17(2):216–32. doi: 10.1074/mcp.RA117.000470 (PMC5795388; doi:10.1074/mcp.RA117.000470)
Supplement: Supplemental Data [file supp_RA117.000470_133922_0_supp_23978_fzffwf.zip › spectra_annotation/mito_spectra_annotation/361-1-12-1-8-1.pdf]

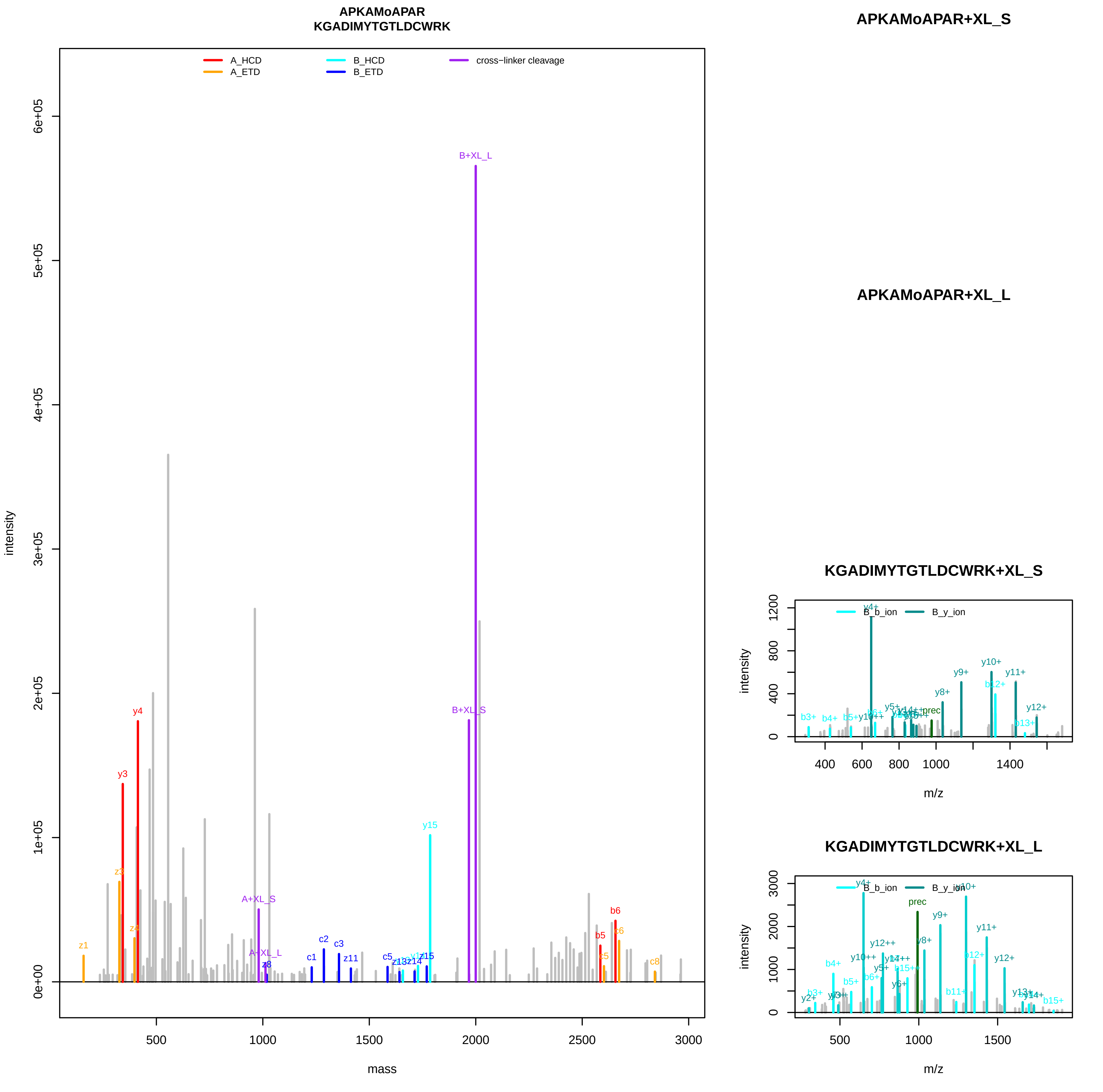
APKAMoAPAR
KGADIMYTGTLDCWRK
A_HCD
A_ETD
B_HCD
B_ETD
cross−linker cleavage
B+XL_L
B+XL_S
A+XL_S
A+XL_L
y4
y3
z3
z4
z1
b5
b6
c5
c6
c8
y15
c1
c2
c3
z11
c5
z13
z14
z15
z8
intensity
mass
APKAMoAPAR+XL_S
APKAMoAPAR+XL_L
KGADIMYTGTLDCWRK+XL_S
B_b_ion
B_y_ion
m/z
KGADIMYTGTLDCWRK+XL_L
prec
